# Association of Amount of Weight Lost After Bariatric Surgery With Intracranial Pressure in Women With Idiopathic Intracranial Hypertension

**DOI:** 10.1212/WNL.0000000000200839

**Published:** 2022-09-13

**Authors:** Susan P. Mollan, James L. Mitchell, Andreas Yiangou, Ryan S. Ottridge, Zerin Alimajstorovic, David M. Cartwright, Simon J. Hickman, Keira A. Markey, Rishi Singhal, Abd A. Tahrani, Emma Frew, Kristian Brock, Alexandra Jean Sinclair

**Affiliations:** From the Birmingham Neuro-Ophthalmology (S.P.M.), University Hospitals Birmingham NHS Foundation Trust, Queen Elizabeth Hospital; Metabolic Neurology (J.L.M., A.Y., Z.A., K.A.M., A.J.S.), Institute of Metabolism and Systems Research, College of Medical and Dental Sciences, University of Birmingham; Department of Neurology (J.L.M., A.Y., A.J.S.), University Hospitals Birmingham NHS Foundation Trust, Queen Elizabeth Hospital; Centre for Endocrinology (J.L.M., A.Y., A.A.T., A.J.S.), Diabetes and Metabolism, Birmingham Health Partners; Birmingham Clinical Trials Unit (R.S.O.); Institute of Metabolism and Systems Research (Z.A., D.M.C., A.A.T., A.J.S.), College of Medical and Dental Sciences, University of Birmingham, Birmingham, United Kingdom; Department of Neurology (S.J.H.), Royal Hallamshire Hospital, Sheffield, United Kingdom; Upper GI Unit and Minimally Invasive Unit (R.S.), Birmingham Heartlands Hospital, University Hospitals Birmingham NHS Foundation Trust, Birmingham; Institute of Cancer and Genomic Sciences (R.S.), University of Birmingham; Department of Endocrinology (A.A.T.), University Hospitals Birmingham NHS Foundation Trust, Queen Elizabeth Hospital; Health Economics Unit (E.F.), Institute of Applied Health Research, University of Birmingham; and Cancer Research UK Clinical Trials Unit (K.B.), University of Birmingham, Birmingham, United Kingdom.

## Abstract

**Background and Objectives:**

The idiopathic intracranial hypertension randomized controlled weight trial (IIH:WT) established that weight loss through bariatric surgery significantly reduced intracranial pressure when compared with a community weight management intervention. This substudy aimed to evaluate the amount of weight loss required to reduce intracranial pressure and to explore the effect of different bariatric surgical approaches.

**Methods:**

IIH:WT was a multicenter randomized controlled trial. Adult women with active idiopathic intracranial hypertension and a body mass index ≥35 kg/m^2^ were randomized to bariatric surgery or a community weight management intervention (1:1). This per-protocol analysis evaluated the relationship between intracranial pressure, weight loss, and the weight loss methods. A linear hierarchical regression model was used to fit the trial outcomes, adjusted for time, treatment arm, and weight.

**Results:**

Sixty-six women were included, of whom 23 had received bariatric surgery by 12 months; the mean age was 31 (SD 8.7) years in the bariatric surgery group and 33.2 (SD 7.4) years in the dietary group. Baseline weight and intracranial pressure were similar in both groups with a mean weight of 119.5 (SD 24.1) and 117.9 (SD 19.5) kg and mean lumbar puncture opening pressure of 34.4 (SD 6.3) and 34.9 (SD 5.3) cmCSF in the bariatric surgery and dietary groups, respectively. Weight loss was significantly associated with reduction in intracranial pressure (R^2^ = 0.4734, *p* ≤ 0.0001). Twenty-four percentage of weight loss (weight loss of 13.3 kg [SD 1.76]) was associated with disease remission (intracranial pressure [ICP] ≤ 25 cmCSF). Roux-en-Y gastric bypass achieved greater, more rapid, and sustained ICP reduction compared with other methods.

**Discussion:**

The greater the weight loss, the greater the reduction in ICP was documented. Twenty four percentage of weight loss was associated with disease remission. Such magnitude of weight loss was unlikely to be achieved without bariatric surgery, and hence, consideration of referral to a bariatric surgery program early for those with active idiopathic intracranial hypertension may be appropriate.

**Trial Registration:**

ClinicalTrials.gov Identifier: NCT02124486; ISRCTN registry number ISRCTN40152829; doi.org/10.1186/ISRCTN40152829.

**Classification of Evidence:**

This study provides Class II evidence that weight loss after bariatric surgery results in reduction in intracranial pressure in adult women with idiopathic intracranial hypertension. This study is Class II because of the use of a per-protocol analysis.

Idiopathic intracranial hypertension (IIH) is characterized by raised intracranial pressure (ICP) that causes chronic headaches and papilledema with the risk of permanent visual loss.^1-3^ Both the incidence and prevalence of IIH in preceding decades has increased,^4-6^ linked with the worldwide obesity epidemic.^7^

Modest weight gain (approximately 5% were people with obesity) is associated with an increased risk of developing IIH, and in those people who do not have obesity, recent weight gain is a risk.^8,9^ Above a body mass index (BMI) threshold of 30 kg/m^2^, the incidence of the disease has been shown to exponentially increase as the BMI increases.^5^ Increased body weight, particularly visceral adiposity, drives the disease.^10,11^ Recent research has shown that IIH has metabolic underpinnings, and patents with IIH have been shown to have unique androgen signatures when compared with people of the same sex, age, and body weight.^12-14^ Those with IIH were more likely to have insulin resistance and hyperleptinemia compared with matched controls.^15^ IIH adipose has a different transcriptional profile compared with that of matched controls, which predisposes them to lipolysis and weight gain. In addition, adipose tissue metabolism in patients with IIH has differential substrate utilization in keeping with tissue primed for lipolysis and weight gain.^15^

Weight loss is known to be an effective treatment for IIH, with a reduction in body weight of between 3% and 15% inducing disease remission, defined by ICP normalization and papilledema resolution.^16,17^ However, maintaining weight loss is challenging, and in general, lost weight will be regained over a 2- to 5-year period.^18^ For those with IIH, this could result in multiple recurrences, with the risk of sight loss and chronic headaches.^8,15,19^ Sustained weight loss in IIH is therefore necessary to modify the disease and prevent relapses.^1,19^ However, the amount of weight loss required to reduce ICP has not been established and has been highlighted as a gap in knowledge with direct clinical relevance.^20^

The IIH:weight trial (IIH:WT) was the first randomized clinical trial to evaluate the efficacy of bariatric surgery compared with a community weight management intervention among patients with active IIH.^21^ Reductions in ICP, disease remission, and superior quality of life outcomes at 2 years were reported when compared with a community weight management intervention (CWI).^22^ The aim of this per-protocol analysis of IIH:WT was to evaluate the amount of weight loss required to reduce ICP and investigate whether there were differences between weight loss surgery methods.

## Methods

IIH:WT was a 5-year randomized, controlled, parallel-group, multicenter trial.^20^ IIH:WT recruited participants at 5 UK National Health Service (NHS) hospitals between July 25 2014 and May 25 2017. The trial protocol detailed inclusion and exclusion criteria, in which those who were pregnant or planning pregnancy during the course of the trial were excluded.^21^ The sample size calculation and considerations, randomization methods, and outcome measures have been published.^21,22^ Written informed consent was obtained from all participants (or guardians of participants) in the study. Women aged between 18 and 55 years, with a BMI ≥35 kg/m^2^, who had failed to lose or maintain weight loss, and who had a clinical diagnosis of active IIH^23^ were randomized into a 1:1 ratio to either WeightWatchers, the chosen CWI, or a bariatric surgery pathway, stratified by the use or nonuse of acetazolamide. However, not everyone received their treatment allocation. This per-protocol analysis was conducted for the primary outcome as part of a planned secondary analysis. Six participants in the surgery arm did not receive bariatric surgery based on personal choice, and no participants were medically declined for surgery. The per-protocol analysis population was defined as the bariatric surgery arm where participants had undergone surgery within 12 months of randomization and the diet weight management arm where participants did not undergo bariatric surgery by 12 months.

The outcome measures included ICP as measured by lumbar puncture opening pressure; anthropometrics; and perimetric mean deviation using Humphrey 24-2 Swedish Interactive Thresholding Algorithm central threshold automated perimetry. Optic nerve head swelling was assessed using spectral domain optical coherence tomography (Spectralis, Heidelberg Engineering) using both the global peripapillary retinal nerve fiber layer thickness and the disc volume central thickness measurements. Headache was evaluated using the headache impact test-6 disability questionnaire (HIT-6), severity scores (numeric rating scale 0 to 10 maximum), and frequency (days per month). The analysis was completed on received data only when every effort was made to follow-up participants, even after protocol violation, to minimize potential for bias. Evaluations included were at baseline, 2 weeks (for those in the bariatric surgery arm only), 12 months, and 24 months. The study protocol and statistical analysis plan were published.^21^

### Statistical Analysis

Statistical analysis was performed in R v3.6.3 (R Foundation for Statistical Computing, Austria). Data were reported with mean values and SD (median values and ranges for non-normal data) and 95% CIs where appropriate. Missing data were not imputed. Statistical significance was determined by ordinary 1-way analysis of variance with the Tukey multiple comparisons test (mean and SEM). Hierarchical linear regression models were used to analyze repeated measures of the primary and secondary outcomes and estimate differences adjusted for baseline values. In these models, population-level effects (also known as fixed effects) comprised the intercept, time as a factor variable, and the 2-way interaction of treatment arm and time as a factor variable to model changing treatment effects over time. Group-level effects (also known as random effects) comprised patient-level adjustments to the intercept. The threshold for statistical significance was prespecified at *p* = 0.05.

### Data Availability

Individual participant data, after anonymization, will be made available, along with the study protocol, statistical analysis plan, and consent forms. On reasonable requests, data beginning at 12 months and ending 3 years after publication of this article will be provided to researchers whose proposed use of the data is approved by the original study chief investigator. Proposals should be made to the corresponding author and requesters will need to sign a data access agreement.

### Standard Protocol Approvals, Registrations, and Patient Consents

Ethical permission for the IIH:WT was obtained from the National Research Ethics Committee West Midlands (14/WM/0011). The trial was registered at ClinicalTrials.gov (Identifier: NCT02124486) and ISRCTN (registry number ISRCTN40152829; doi.org/10.1186/ISRCTN40152829). Written informed consent was obtained from all participants. All necessary patient/participant consent has been obtained, and the appropriate institutional forms have been archived.

## Results

**Figure 1:**
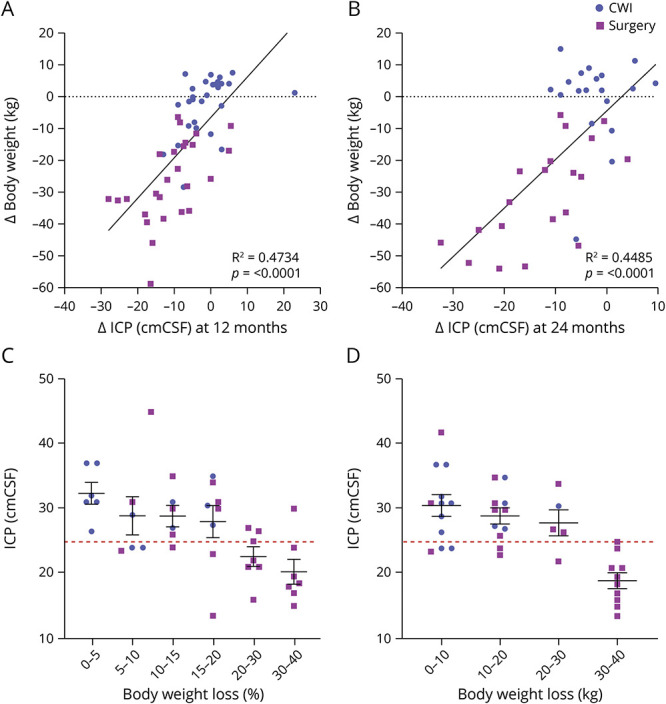
Reduced Body Weight Significantly Correlates With Reduced ICP (A, B) Linear regression analysis plotting change in body weight against change in ICP at 12 and 24 months postbaseline. (C, D) ICP levels of patients categorized according to percentage and absolute weight loss at 12 months since baseline measurements. The dashed red line indicates the idiopathic intracranial hypertension diagnostic threshold of an ICP >25 cmCSF. Data presented as mean ± SEM. Statistical significance was determined by ordinary 1-way analysis of variance with the Tukey multiple comparisons test. CWI = community weight management intervention; ICP = intracranial pressure.

Sixty-six women were recruited. The study population experienced active disease, as evidenced by the mean ICP of 32.5 (SD 7.8) cmCSF (Table 1). In this planned per-protocol analysis, 20 women who had undergone bariatric surgery were compared at 12 months with 43 who were receiving lifestyle weight management advice (either through WeightWatchers or as part of the bariatric surgery pathway). At baseline, 18 of the 66 were taking acetazolamide; by 12 months, 1 patient in the bariatric surgery arm was still taking 500 mg daily and 8 of the 43 remained on acetazolamide (mean dose 844 mg [SD 498.9]). At both 12 and 24 months, weight and BMI reductions were greater in the bariatric surgery group than in the diet weight management group (eTables 1 and 2, links.lww.com/WNL/C169). For the percentage of weight change and the percentage of excess weight loss, the mean difference (SEM) (95% CI) between those who underwent surgery and those who did not undergo surgery at 12 months was −18.3% (1.9); (−22.1, −14.6), *p* < 0.001 (Figure 1) and −46.4% (4.9); (−56.1, −36.7), *p* < 0.001, respectively. The 24-month results were similar: −23.6% (2.1); (−27.8, −19.4), *p* < 0.001 and −61.6% (5.5); (−72.3, −50.8), *p* < 0.001, respectively, with the greatest changes seen in those who underwent bariatric surgery (Figure 1).

**Table 1:**
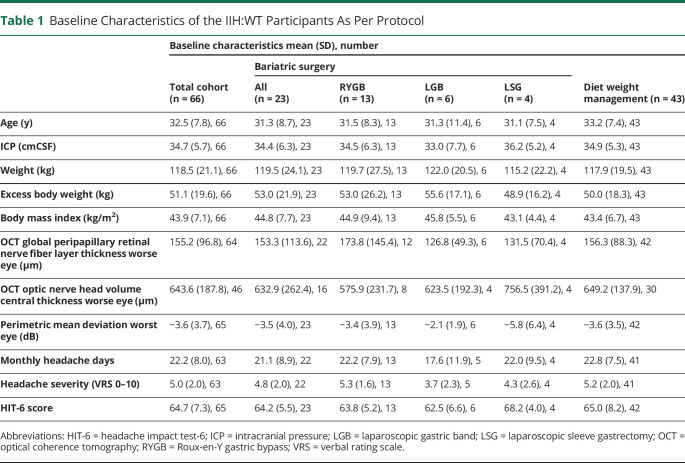
Baseline Characteristics of the IIH:WT Participants As Per Protocol

**Figure 2:**
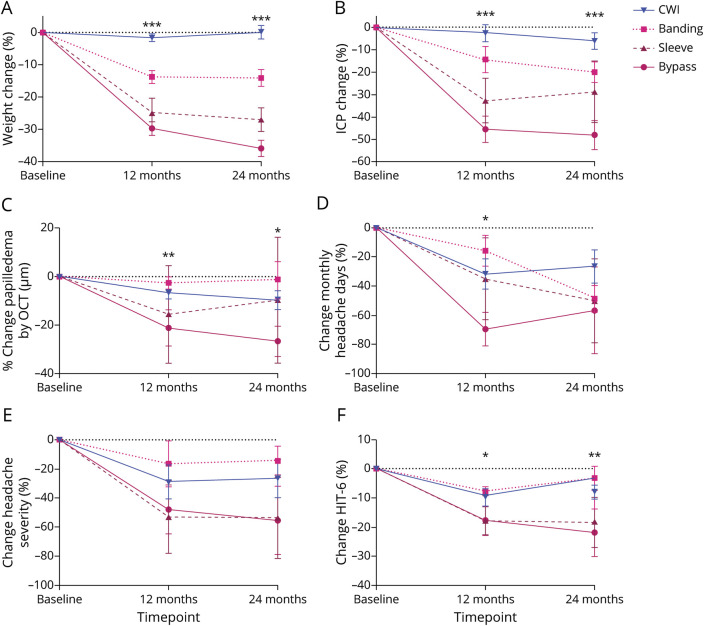
Surgical Intervention is Significantly More Efficacious at Lowering Body Weight and ICP Than Diet Weight Loss Intervention Percentage change in diet and surgery groups at baseline, 12-month, and 24-month timepoints for (A) body weight; (B) intracranial pressure; (C) papilledema as measured by OCT volume central thickness; (D) monthly headache days; (E) headache severity; and (F) HIT-6 score; data presented as mean ± SEM. Statistical significance was determined by hierarchical regression modeling in accordance with per-protocol analysis. ***p < 0.001. CWI = community weight management intervention; HIT-6 = headache impact test-6; ICP = intracranial pressure; OCT = optical coherence tomography.

Correlation analysis showed that in the total study population, reducing body weight significantly correlated with reducing ICP at both 12 and 24 months (R^2^ = 0.47, *p* < 0.0001 and R^2^ = 0.45, *p* < 0.0001, respectively) (Figure 2, A and B). To further understand this relationship of weight change and ICP levels, weight loss outcomes were summarized by ICP categories (Table 2), and ICP outcomes were summarized by weight loss categories (Table 3). Only those in the bariatric surgery arm managed to achieve sufficient weight loss (in kilograms), which resulted in a fall of ICP below the IIH diagnostic threshold of an ICP ≤25 cmCSF (Figure 2, C and D; Table 3). The mean weight loss required for an ICP ≤25 cmCSF at 12 months was −13.3 kg (1.76) (a 24% decrease in body weight) (Table 2). For ICP to be ≤30 cmCSF, the mean weight loss was −9.94 kg (1.34) (18% decrease in weight) (Figure 2; Table 2). An increased weight loss conferred a proportionally greater drop in ICP (5% weight loss led to a 10% [−4.1 cmCSF] decrease in ICP, 10% weight loss led to a 14% [−4.4 cmCSF] decrease in ICP, and 20% weight loss led to a 26% [−10.2 cmCSF] decrease in ICP) (Table 3).

**Table 2:**
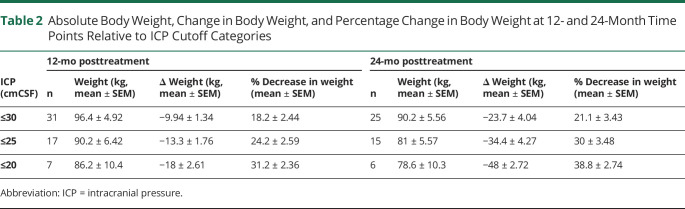
Absolute Body Weight, Change in Body Weight, and Percentage Change in Body Weight at 12- and 24-Month Time Points Relative to ICP Cutoff Categories

**Table 3:**
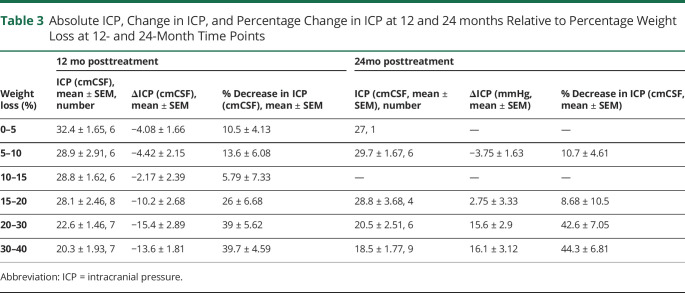
Absolute ICP, Change in ICP, and Percentage Change in ICP at 12 and 24 months Relative to Percentage Weight Loss at 12- and 24-Month Time Points

**Figure 3:**
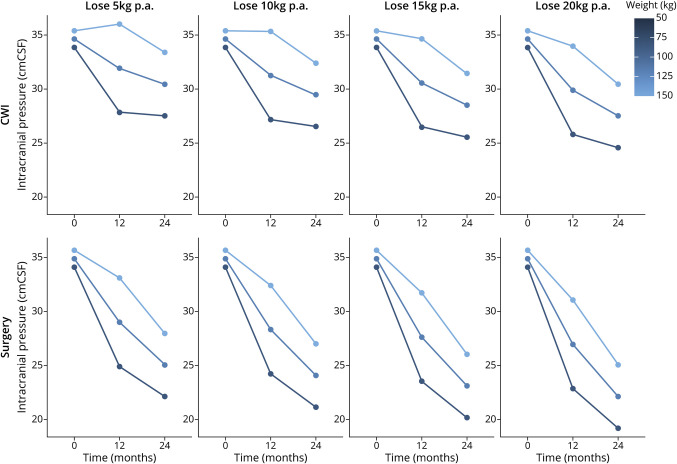
Model-Generated Expected ICP Outcomes for Three Notional Participants With Baseline Weights of 150 kg (Top Line), 120 kg (Middle) and 90 kg (Bottom), Allocated to Each Treatment Arm Under 4 Different Weight Loss Scenarios The expected ICP values are predicted by a hierarchical model fit to the trial outcomes, adjusted for time, intervention, and contemporaneous weight. CWI = community weight management intervention; ICP = intracranial pressure; p.a. = per annum.

The relationship between ICP and weight change was further explored in a hierarchical model to fit the trial outcomes, adjusted for time, intervention, and contemporaneous weight to predict expected ICP values from weight loss. This modeling demonstrated that greater reduction in ICP was predicted with greater weight loss (Figure 3). The effect on ICP further improved between 12 and 24 months as the participants continued to lose weight. For expected ICP values to meet or cross the threshold for normal, at 25cmCSF within 2 years, the model predicted that a patient with a baseline weight of 150 kg would have to have been allocated to the surgery arm and achieved a weight of 110 kg. This predictive modeling showed that those with a higher starting weight needed to lose more weight to meaningfully reduce ICP. This model also demonstrated that among those in the diet arm, if no or little weight loss was achieved in those with a high baseline weight, an increase in ICP would be expected (Figure 3).

Roux-en-Y gastric bypass (RYGB) surgery was the most common surgery performed (n = 13) and proved to be the most successful weight loss method, compared with gastric banding, gastric sleeve, and dietary intervention, recording a reduction at 12 months of −34.9 kg from baseline (adjusted mean difference [95% CI]: −34.9 [−40.0, −29.8]; *p* < 0.001) (eTable 3, links.lww.com/WNL/C169). The effect size increased with a mean of −42.5 kg weight loss between baseline and 24 months (adjusted mean difference [95% CI]: −42.5 [−47.9, −37.1]; *p* < 0.001). (eTable 4) At both 12 and 24 months, the reduction in ICP was greater in the bariatric surgery group than in the diet weight management group (*p* < 0.001) (eTable 5; Figure 1). ICP in the bariatric surgery arm 2 weeks postsurgery showed that the mean ICP (SD) decreased from 34.7 (5.7) cmCSF at baseline to 26.9 (8.1) cmCSF (*p* < 0.001).^22^ RYGB recorded the greatest reduction in ICP with the adjusted difference in ICP of −14.4 cmCSF between baseline and 12 months (adjusted mean difference [95% CI]: −14.4 [−18.1, −10.7]; *p* < 0.001) (eTable 3). ICP at 24 months was recorded to have fallen further (Figure 1B) with the difference between baseline and 24 months with RYGB-adjusted difference of −17.5 (SD 2.0) cmCSF; (adjusted mean difference [95% CI]: −21.4, −13.6; *p* < 0.001) (eTable 3).

Significant reductions in measures of papilledema and headache outcomes were observed with all surgical approaches, with the greatest benefit seen with RYGB (eTable 3, links.lww.com/WNL/C169). RYGB was superior to diet weight management at 12 and 24 months with significant reductions in papilledema, as measured by the optic nerve head volume central thickness, *p* < 0.01 and *p* < 0.04 respectively (eTable 5; Figure 1). There was a significant reduction in monthly headache days at 12 months (*p* < 0.05) (eTable 5; Figure 1), but there was no difference at 12 or 24 months in headache severity score (eTable 5; Figure 1). The percentage change in the headache impact test (HIT)-6 score was significant between the 2 arms at both 12 (*p* = 0.019) and 24 months (*p* = 0.003) (eTable 5; Figure 1).

This study provides CII evidence that weight loss after bariatric surgery results in reduction in intracranial pressure in adult women with idiopathic intracranial hypertension. This study is Class II because of the use of a per-protocol analysis.

## Discussion

In this per-protocol analysis of IIH:WT, we have demonstrated the extent of weight loss was directly associated with, and predicted, reduction in ICP. In women with active IIH and a BMI >35 kg/m^2^, the amount of weight loss required to normalize the ICP to a level of ≤25 cmCSF was 24% of baseline body weight. To achieve this, it was generally required that the patient be allocated to the bariatric surgery arm. RYGB was the superior procedure for weight loss, ICP reduction, and improvement in both papilledema measures and headache outcomes when compared with the other surgical procedures.

This analysis shows that greater weight loss was associated with greater reductions in ICP, which may not be surprising, considering the previous medical literature linking obesity and IIH.^5,8,9^ In a previous study, a very low-energy diet (≤425 kcal/d) for 3 months induced 15% weight loss and lowered ICP significantly (mean 8.0 [SD 4.2] cm CSF, *p* < 0.001). Over the course of the study, improvements in papilledema and visual function and decreased headache frequency and severity with concomitant reduction in analgesic use were noted.^17^ However, the amount of weight loss required to normalize ICP (i.e., to a level of or less than 25 cmCSF) had not previously been established. In the IIH:WT, there was a significant difference in the primary outcome of ICP at 12 months in those who underwent bariatric surgery, when compared with the dietary intervention, with an enduring effect at 24 months.^22^ When the trial outcomes from all participants were modeled in this study (Figure 3), this demonstrated that greater reduction in ICP was predicted with greater weight loss. Those with a higher starting weight needed to lose more weight to meaningfully reduce ICP. The model demonstrated that in the diet group, if no or little weight loss was achieved in those with a high baseline weight, an increase in ICP would be expected. Therefore, caution should be applied when exposing women with IIH to repeat lifestyle interventions, given the risk of recurrence of their disease and the potential compound effect on repeated episodes of papilledema on the optic nerve health. To cross the ICP lumbar puncture opening pressure threshold of ≤25 cmCSF for all weight loss scenarios, the model predicted that allocation to the bariatric surgery pathway was needed. Hence, clinicians should have low thresholds to refer for bariatric surgery services and not delay weight loss treatment intensification in those who could not achieve adequate weight loss previously or had weight regain. In addition, it is important to consider the effect of weight loss beyond the immediate IIH outcomes. We have shown previously that patients with IIH have an increased risk of cardiovascular disease compared with women with similar BMI.^5^ Previous studies showed that bariatric surgery is associated with reduction in CVD and mortality in patients with obesity.^24^ This further emphasizes the importance of not delaying bariatric surgery unnecessarily in women with IIH.

Of note was that at 2 weeks postoperatively, there was a significant reduction in ICP. This is consistent with other studies that showed rapid improvements in obesity complications within 3–4 weeks after bariatric surgery, particularly in type 2 diabetes.^25^ There are multiple plausible mechanisms underpinning such quick improvement in ICP including the pre surgical liver shrinkage low-calorie diet, weight loss, and the changes in gut hormones that occur following gastric bypass and sleeve gastrectomy. Our group has previously shown a potential role for GLP-1 receptor agonist in reducing ICP; hence, this could be a possible mechanism in patients who underwent RYGB or sleeve gastrectomy, considering their effect on GLP-1 levels.^24,26,27^ The mechanism for this reduction could be debated as an influence of the weight lost in the perioperative period, a direct metabolic effect from gut neuropeptides and their action on the choroid plexus, or a combination of both. What is clear is that for those who require a more expedient reduction in ICP, bariatric surgery could potentially be an acute treatment option for those with IIH in some healthcare settings.^11^

There are several limitations of the analysis, which include the small numbers in each of the bariatric surgery types, and while we have presented the favorable results with RYGB, no specific type of surgery should be recommended over another because this requires further investigation. It may be important to note that those who did not receive surgery had 2 types of weight management programs, one being the weight watchers and the other within the bariatric surgery program, and this could have influenced the results in this group, with the hospital-based weight management program being more structured. In addition, when considering bariatric surgery as a treatment option for IIH, it may not be suitable for everyone. We recommend careful counseling by experts to discuss the side-effect profile, lifelong changes, and the permanent nature of the surgery.

Although bariatric surgery in IIH:WT had high upfront costs,^28^ it was more cost-effective with time, both saving money and improving the quality of life at the 5-, 10-, and 15-year time horizons considered.^29^ This analysis provides further evidence for considering different types of weight loss methods, supporting bariatric surgery as a management option to be considered in women with active IIH. Bariatric surgery procedures vary in their weight loss outcomes their effect on obesity complications and weight loss. Unfortunately, our study was limited by being too small to determine which procedure is best for women with IIH. However, our findings that RYGB resulted in greater weight loss are consistent with the literature.^30^ The choice of which procedure to perform should be based on a shared decision-making process between the patient and the surgeon, considering the potential benefits, harms, and complications if any are present.

In women with active IIH and a BMI >35 kg/m^2^, the amount of weight loss required to normalize the ICP to a level of ≤25 cmCSF is 24% of baseline body weight. This is unlikely to be achieved by dietary interventions alone, and early referral to a bariatric surgical pathway should be considered.

## Glossary

BMI: body mass index
CWI: community weight management intervention
HIT-6: headache impact test-6
ICP: intracranial pressure
IIH: idiopathic intracranial hypertension
RYGB: Roux-en-Y gastric bypass
WT: weight trial
